# ADHD polygenic risk predicts neural signatures of cognitive control: Evidence from midfrontal theta dynamics

**DOI:** 10.1038/s41398-026-03938-2

**Published:** 2026-03-31

**Authors:** Ümit Aydin, Ziye Wang, Máté Gyurkovics, Amy Tong, Grace Cullen, Sumayyah Ahmed, Jason Palmer, Gráinne McLoughlin

**Affiliations:** 1 School of Psychology & Clinical Language Sciences, University of Reading, Reading, United Kingdom; 2 Social, Genetic & Developmental Psychiatry Centre, Institute of Psychiatry, Psychology & Neuroscience, King’s College London, London, United Kingdom; 3 Department of Biological and Experimental Psychology, School of Biological and Behavioural Sciences, Queen Mary University of London, London, United Kingdom; 4 School of Psychology, University of East Anglia, Norwich, United Kingdom; 5 Department of Psychological Medicine, Institute of Psychiatry, Psychology & Neuroscience, King’s College London, London, United Kingdom; 6 Faculty of Biology, Medicine and Health, University of Manchester, Manchester, United Kingdom; 7 School of Mathematical and Data Sciences, West Virginia University, Morgantown, WV USA

**Keywords:** Clinical genetics, Human behaviour, ADHD

## Abstract

Cognitive control mechanisms ensure goal-directedness in behaviour. Difficulties in cognitive control are well-established in conditions such as attention-deficit/hyperactivity disorder (ADHD) and autism. On the neural level, midfrontal theta (4–8 Hz) activity has emerged as a reliable correlate of cognitive control processes. Previous findings showed alterations in theta-based signals in both ADHD and autism, most notably an increase in the variability of theta phases across trials in cognitive control tasks, which was predictive of increased response time variability (RTV) as well. Crucially, recent work on twin studies has provided strong evidence for the genetic underpinning of these associations. Here, for the first time, we investigated whether polygenic scores (PGS) for ADHD and autism can predict RTV and EEG-derived theta-based measures of cognitive control in 454 participants. We found that PGS for ADHD, but not autism, accounted for a significant proportion of the variance in theta phase variability, captured via inter-trial coherence (ITC), in the well-standardised arrow-flanker task (2.5% of total variance, corresponding to 3.3% of the reliable variance). Furthermore, theta-ITC showed excellent test-retest reliability in our sample, indicating psychometric robustness, which in turn, further strengthens our findings. These results provide robust evidence linking genetic risk to neural measures and suggest that core dysregulation of the temporal coordination of control processes in ADHD is under genetic influence.

## Introduction

Cognitive control, a fundamental set of mental processes crucial for goal-directed behaviour in everyday life, orchestrates the prioritisation of task-relevant information while suppressing irrelevant distractions [1]. Cognitive control mechanisms are implicated in various psychopathologies [2–6], with particular relevance to neurodevelopmental conditions such as attention-deficit/hyperactivity disorder (ADHD) and autism spectrum disorder (ASD) [7–15].

A robust neurophysiological marker of cognitive control has emerged across diverse experimental paradigms: midfrontal theta or frontal midline theta (FMθ), a 4–8 Hz oscillatory activity observed over medial frontal recording sites. FMθ is consistently correlated with cognitive control processes, manifesting as brief bursts of theta oscillations time-locked to relevant stimuli and/or responses [16–18]. FMθ activity emerges during tasks requiring the maintenance of goal-directedness in the face of interference, serving as a neural marker of cognitive control engagement [19, 20]. Importantly, alterations in FMθ activity (including event-related potentials/ERPs, representing time-locked FMθ) have been repeatedly associated with ADHD and autism, aligning with the cognitive control difficulties observed in these conditions [21–23].

The connection between neural oscillations and behaviour is further illuminated by the relationship between FMθ and reaction time variability (RTV), a measure of performance consistency. Specifically, cross-trial variability in FMθ phase during cognitive tasks - which reflects inconsistency in the timing of neural activity across repeated instances of task performance - has been consistently linked to RTV [24–29]. Such neural variability suggests a lack of precise temporal coordination in cognitive control processes [4, 15]. This finding takes on particular significance in the context of ADHD, where increased RTV is recognised as a domain-general behavioural characteristic of the condition [30–33].

Our previous twin studies, using genetic multivariate model fitting, have provided compelling evidence for the genetic underpinnings of these relationships. In two independent studies, we demonstrated significant genetic associations between variability in both theta phase and reaction time and ADHD in both adolescence and young adulthood [15, 17]. Crucially, these relationships remained stable across time, indicating a persistent core dysregulation of the temporal coordination of control processes in individuals with childhood ADHD symptoms [15]. Our previous work additionally replicated previous findings showing strong phenotypic overlap between RTV and autism [34] and indicated a moderate to strong genetic overlap, especially between RTV and autism in adulthood [15].

We further found that error processing, as indexed by the amplitude of the error positivity (Pe), was altered in both ADHD and Autism, with a strong genetic contribution to these alterations. The Pe identified in this work exhibited a frontocentral distribution, consistent with time- and phase-locked theta-range activity and suggesting functional integration with FMθ dynamics during error processing [15]. This topography aligns with extensive prior evidence that the Pe can show an early frontocentral distribution [35, 36]. These findings underscore the robust association between FMθ-indices of cognitive control, behavioural variability (RTV) and provide a compelling framework for understanding the neural underpinnings of cognitive control deficits in ADHD and autism, suggesting shared genetic influences on both neural markers of cognitive control and disorder symptomatology.

The genetic architecture of both ADHD and autism is characterised by polygenic variation, rare structural variants, and epigenetic effects [37, 38]. Both conditions show high heritability, with ADHD estimated at 70–80% [38] and ASD at around 80% [39]. An approach to index genetic risk for these disorders is via the cumulative effect of frequent genetic variants using a polygenic score (PGS). Examination of the relationship between disorder-specific PGS and behavioural and neurophysiological markers allows us to bridge the gap between genetic risk and specific brain function indicators, including measures of cognitive control. Brain function markers that exhibit familial patterns and heritability may serve as valuable endophenotypes - quantifiable traits that may bridge the gap between genes and observable characteristics - potentially offering a more nuanced approach to deciphering the complex genetic underpinnings of neurodevelopmental conditions [40]. These neurophysiological and behavioural markers of cognitive control provide quantifiable indices of cognitive processes and their temporal dynamics, offering insight into how genetic risk may manifest in brain function and behaviour. By linking PGS to FMθ-indices of cognitive control and behavioural variability (RTV), we can better understand the pathways through which genetic liability influences cognitive control processes implicated in ADHD and ASD.

Building on this evidence, we aim to test whether PGS for ADHD and autism can predict these neurophysiological measures of cognitive control and behavioural variability [37, 41]. Specifically, in a young adult sample, we examine if PGS for these conditions are associated with increased FMθ phase variability, attenuated Pe amplitude, and greater RTV - measures that previously showed genetic overlap with ADHD and autism in genetic multivariate twin model fitting in the current sample [15, 17]. In our previous study using the same sample, we found significant phenotypic correlations between theta ITC and RTV (r = −0.39), and between both measures and ADHD symptoms (ITC-childhood ADHD: r = −0.23; RTV-childhood ADHD: r = 0.20) and autism symptoms (ITC-childhood autism: r = −0.07; RTV-childhood autism: r = 0.13). Importantly, substantial genetic correlations underpinned these relationships (ITC-RTV: rₐ = −0.68; ITC-childhood ADHD: rₐ = −0.44; RTV-childhood ADHD: rₐ = 0.34; RTV-childhood autism: rₐ = 0.34), providing the foundation for testing whether polygenic scores would show similar associations with these neural and behavioural measures [15]. This approach allows us to bridge the gap between molecular genetic liability for neurodevelopmental conditions and specific neural and behavioural phenotypes associated with cognitive control.

To enhance the robustness of our findings, we address an important methodological consideration. Psychometrically sound measures are essential for the development of brain biomarkers in mental health research [42–47]. Recognising this, we collected data at two time points from an age-matched sample to calculate the test-retest reliability of the neurophysiological and reaction time measures. This approach allows us to estimate the proportion of reliable variance in our measures that is explained by PGS.

## Methods and materials

The publication is part of the analysis that was pre-registered https://osf.io/s5d7t, prior to accessing the polygenic data.

### Sample

The total sample consisted of 566 twins (271 males) with an average age of 22.43 ± 0.96 (range: 20.4 - 25.1) years. Participants were recruited from the Twins’ Early Development Study (TEDS), a longitudinal community-based twin cohort [48, 49]. Recruitment specifically targeted individuals with elevated levels of ADHD and/or autistic traits assessed in childhood and adolescence, alongside individuals with lower trait levels, in order to create a sample enriched for neurodevelopmental trait variation: see Supplement and [50, 51]. Clinical assessments were conducted during IDEAS using the Diagnostic Interview for ADHD in Adults 2.0 (DIVA-2) and the Autism Diagnostic Observation Schedule-2 (ADOS-2) to assess current DSM-5 symptoms. Based on these measures, 111 participants met criteria for ADHD and 47 for autism, including 16 that met the criteria for both ADHD and autism [15]. Out of 566, DNA samples were collected from 454 participants previously as part of TEDS. All participants were of white ethnic origin, based on parental self-report from the TEDS first contact questionnaire. Ethnicity was recorded using broad categories in line with national classifications at the time, and ‘White’ was not further sub-classified (see [48]). The test-retest reliability study involved a distinct sample of 21 participants (20 females, 1 male; mean age = 20.14 years, SD = 1.51, range = 19–24 years) recruited independently from the main study cohort. Exclusion criteria included any diagnosed neurological or psychiatric conditions.

Ethical approval for the study was received from King’s College London Psychiatry, Nursing and Midwifery Research Ethics Subcommittee (IDEAS: RESCMR-16/17-2673; TEDS: 05/Q0706/228 and PNM/09/10-104) and all participants signed informed consent forms prior to participation. All methods were performed in accordance with the relevant guidelines and regulations.

### EEG acquisition and cognitive task

EEG data were acquired as part of the Individual Differences in EEG in Young Adults Study (IDEAS) using a 64-channel wireless EEG system (Cognionics, San Diego, CA) with Ag/AgCl electrodes at a sampling rate of 500 Hz. Each participant in the main sample completed four cognitive tasks and a resting state run in a single session. Tasks were delivered in counterbalanced order across the whole sample, but the order was kept constant within each twin pair. The order of the tasks did not significantly impact the measures used in this study [15].

This study presents the results from the arrow flanker task, a modified version of the Eriksen flanker task [15, 17, 52]. The task consisted of 10 blocks of 40 trials. Two flankers (black arrowheads above and below the position of a fixation mark) were presented for 100 ms before the central target black arrowhead appeared for an additional 150 ms. Participants had to press a response button with the index finger of the hand corresponding to the direction indicated by the target arrow (left or right). On congruent trials, flanker and target arrowheads pointed in the same direction; on incongruent trials, they pointed in opposite directions. The test-retest reliability study replicated the EEG setup and arrow flanker task of the main study. Participants underwent two sessions, averaging 8.1 days apart.

### Polygenic scores

Polygenic scores calculated from genotypic data were obtained from TEDS. Details of how the polygenic scores were derived are in the supplement and [53, 54]. Each polygenic score is a numerical value that combines information from multiple genetic variations across the genome, with each variation contributing based on its relevance to a particular trait. The specific polygenic score variables used in this study from TEDS were variable PGC2017 for ADHD [41] and iPsych2018 for autism [37] with a threshold of 1. Before further analysis, polygenic scores were adjusted for the first 10 principal components of the genotype data, chip type and batch number via regression and z-standardised [53].

### EEG processing and analysis

EEGLAB and custom MATLAB scripts were used for EEG analysis [55, 56]. Further details of the EEG pre-processing are reported in the supplement and in [15]. EEG data was filtered 1–30 Hz and resampled to 256 Hz. Channels with a correlation of less than 0.4 with their neighbours or above 75 μV (absolute value) for more than 15% of the time were marked as bad channels. An amplitude threshold ( ± 100μV) was used to remove noisy trials and only participants with 20 or more valid trials (per condition) were included in further analysis.

EEG measures used in this study were selected to ensure methodological continuity with our previous twin analysis [15], where these measures showed significant genetic correlations with ADHD and/or autism. These measures were the peak amplitude of error positivity (Pe), and inter-trial coherence (ITC). Pe was calculated from incongruent-incorrect trials, as the peak (maximum) at FCz channel, 100 to 350 ms after the commission of errors [15, 57]. Inter-trial coherence (ITC) was calculated in the theta band using incongruent-correct trials – the condition showing the greatest conflict and need for cognitive control. The precise theta frequency for each participant was identified as the frequency with the highest ITC within the 4–9 Hz range. We extended the theta range to include all participants’ theta activity in the window between stimulus and response, which reached up to 9 Hz. These theta frequencies were then averaged across all participants to determine the overall mean frequency (6.9 Hz), which was used to calculate the ITC for each individual [15].

We also investigated reaction time variability (RTV), calculated as the standard deviation of response latency for incongruent correct trials. As with ITC and the Pe, this measure was selected based on our previous twin study, which demonstrated significant genetic overlap between RTV and both ADHD and autism [15].

### Statistical analysis

Multilevel mixed effect models as implemented in R package lme4 were used for the analysis [58] and a random intercept at the family level was used to control for twin relatedness [59]. Six models were run with one of ITC, Pe or RTV as a dependent variable and PGS of either ADHD or autism as well as sex and age as independent variables. P-values were calculated using the lmerTest package, which uses Satterthwaite’s method to approximate the degrees of freedom [60]. Multiple testing was controlled for all six models using the false discovery rate (FDR) method [61].

Pe and RTV were logarithm transformed (log_e_) and ITC was square (power of two) transformed to ensure normality and scaled (z transformed). Outliers were excluded based on the 2*IQR (interquartile range) criterion. Age of the participants and PGS were also standardised. The same analysis was also performed without any transformation and outlier exclusion and there was no change in the findings in terms of statistical significance.

Test-retest reliability was assessed via the Intraclass correlation coefficient (ICC) and Pearson’s correlation coefficient using SPSS 28. ICC is the standard measure used for reliability testing in most fields, including EEG [62, 63]. The two-way mixed effects model was used, which is the standard model for test-retest reliability. Participant means are assumed random, and the measurement effect is assumed to be zero. Absolute agreement and consistency measures are presented. ICCs quality was considered as poor if ICC < 0.40, moderate if 0.40–0.60, good if 0.60– 0.75, and excellent if higher than 0.75 [62, 63]. For the ERP based measures and mean reaction time (RTM), the average measures ICC statistic was used since the measures are averages across trials. For the variability measures, ITC and RTV, the single measurement statistic was used, since these measures are not averages, but measures of the whole population variability.

The Pearson’s correlation coefficient can be used to correct the variance explained by PGS for reliability. We applied the single correction attenuation formula [64, 65]:

$${r}_{{XY}}\left({corrected}\right)=\frac{{r}_{{XY}}}{\sqrt{{r}_{{YY}}}}$$

where *X* is the PGS, and *Y* is the test variable (ITC, Pe amplitude, RTV), and ${r}_{{YY}}$ is the test-retest correlation (we assume ${r}_{{YY}}$ is positive for a reasonably reliable measure). The corrected correlation squared gives the corrected R², which is an estimate of the upper limit of the true fraction of the measure variance explained by PGS given the degree of reliability of the measures. The corrected R² is then the square of the corrected correlation, ${r}_{{XY}}^{2}/{r}_{{YY}}.$ Here we used the standardised beta coefficients (β_std_) of the fixed effects from linear mixed effect model instead of the correlations ${r}_{{XY}}$.

## Results

**Fig. 1:**
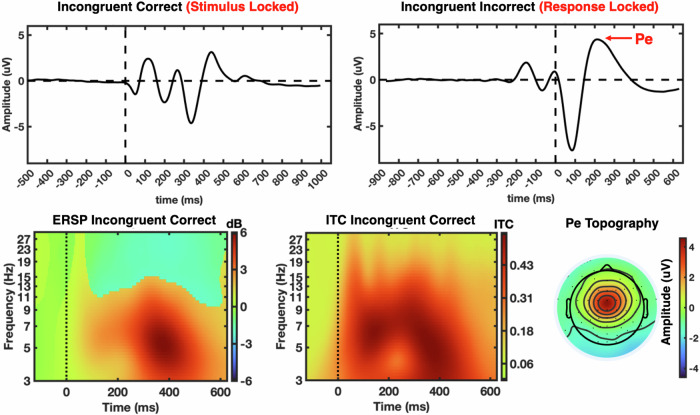
EEG event-related potentials, event-related spectral perturbation (ERSP) and inter-trial coherence (ITC) plots at the FCz channel. The top left shows the grand average waveform for the correctly answered incongruent trials, time locked to stimulus onset, which were used for calculating ITC. The top right is for the incorrectly answered incongruent trials, locked to the response, which were used to calculate Pe (please see the red arrow for the Pe peak). The bottom panel shows the ERSP and ITC time frequency plots (please note the log spaced intervals for frequency) for the correctly answered incongruent trials again time locked to stimulus onset. The topography for the Pe component, calculated from incorrectly answered incongruent trials, is shown at bottom right with black dots indicating electrode positions.

The grand average EEG waveforms as well as event-related spectral perturbation (ERSP) and ITC time frequency plots for the incongruent correct trials (stimulus onset locked), which were used to calculate ITC, and the incongruent incorrect trials (response locked), which were used to calculate Pe amplitude, as well as the Pe topography, are shown in Fig. 1.

The ICC estimates indicated excellent test-retest reliability for ITC and Pe and good reliability for RTV (Table 1). ICCs and Cronbach’s alpha of other EEG and behavioural measures from the arrow flanker task are included in Supplementary Table 1.

**Table 1:** **Test-retest** reliability of ITC, Pe amplitude and RTV calculated from the arrow flanker task.

Variable	ICC (95% CI)	Pearson’s *r* (95% CI)
**ITC**	0.785 (0.540, 0.907)	0.777 (0.520, 0.905)
**Pe amplitude**	0.791 (0.486, 0.915)	0.711 (0.403, 0.874)
**RTV**	0.605 (0.227, 0.821)	0.669 (0.334, 0.854)

*ITC* Inter-trial coherence, Pe Error positivity, *RTV* Reaction time variability.

Table 2 indicates the demographics of the groups (for the complete cases). Generalized linear mixed-effects models were used to assess if age and sex significantly differ between groups. Sex, but not age, was significantly different between the groups. There were more females (57.8%) than males in CG, while the percentage of females in ADHD (38.2%) and autism (23.8%) groups were smaller than males. Age did not have a statistically significant effect in any of the models and the effect of sex was statistically significant only for RTV (β_std_ = −0.25, SE = 0.10, *t*(363.5) = −2.36, *p*_*uncorrected*_ = 0.019) as reported in Table 3.

**Table 2:** Age and sex for each group (Comparison group [CG], ADHD, autism) along with p-values for group differences.

Variable		CG	ADHD	Autism	p-value
**Number of participants**		315	74	21	
**Age**	**Mean (SD)**	22.5 (1.0)	22.2 (1.1)	22.3 (1.0)	0.285
**Sex**	**Male**	133 (42.2%)	46 (62.2%)	16 (76.2%)	0.012
	**Female**	182 (57.8%)	28 (37.8%)	5 (23.8%)	

Demographics for the complete cases only (404 participants), having valid PGS, ITC, Pe, RTV and ADHD and autism diagnoses, are provided. Since the number of complete cases differs for each EEG and behavioural measure (and ADHD/autism diagnosis is not used in the models) the number of observations for each model differs slightly. Please note the total sum of participants in the table is higher than 404 because participants with co-occurring ADHD and autism (six in total) are included in both the ADHD and autism groups.

**Table 3:** Associations between the PGS of ADHD and ITC, Pe and RTV.

	ITC					Pe					RTV				
***Coefficient***	*β*_*std*_	*SE*	*t-value*	*p*	*df*	*β*_*std*_	*SE*	*t-value*	*p*	*df*	*β*_*std*_	*SE*	*t-value*	*p*	*df*
**Intercept**	−0.01	0.08	−0.11	0.911	308.84	0.02	0.08	0.29	0.770	278.52	0.09	0.07	1.27	0.206	281.28
**PGS (ADHD)**	−0.16	0.05	−2.95	0.003*	364.57	0.02	0.05	0.31	0.756	313.80	0.08	0.05	1.47	0.141	306.06
**Age**	−0.00	0.06	0.06	0.995	236.07	−0.03	0.06	−0.55	0.580	220.94	0.07	0.05	1.33	0.184	226.05
**Sex**	0.09	0.10	0.86	0.392	407.45	−0.03	0.11	−0.33	0.744	364.90	−0.25	0.10	−2.36	**0.019**	363.50
	R^2^ = 0.025; corrected R^2^ = 0.033				R^2^ = 0.001; corrected R^2^ = 0.001						R^2^ = 0.026; corrected R^2^ = 0.038				

Uncorrected p-values are given in the table and the p-values that were lower than 0.05 after FDR correction are indicated with *. Only the fixed effect findings are given in the table. SE Standard Error; df degrees of freedom (Satterthwaite’s approximation).

*ITC* Inter-trial coherence, *Pe* Error positivity, *RTV* Reaction time variability.

Table 3 presents the results of linear mixed-effects models examining associations between ADHD PGS and our measures of interest: ITC, Pe amplitude, and RTV. Among these, only ITC showed a statistically significant association with PGS of ADHD (β_std_ = −0.16, SE = 0.05, *t*(364.6) = −2.95, *p*_*uncorrected*_ = 0.003, *p*_*FDR*_ = 0.020) (Table 3). This association remained significant after FDR correction, considering the six fixed effects of PGS reported in Table 3 and Table 4. The full model (including age and sex) accounted for 2.5% of the total variance in ITC (Table 3), which increases to 3.3% of the reliable variance explained when accounting for the reliability of ITC. The PGS alone contributed 2.4% of the total variance (3.1% of reliable variance), indicating that the genetic effect is largely independent of demographic factors (Table 5). Sensitivity analyses indicating that the PGS–ITC association remains significant when approximating population ADHD prevalence, through subsampling ADHD individuals, and when analysing only controls, or only participants with ADHD, as reported in Supplementary Table 9.

**Table 4:** Associations between the PGS of Autism and ITC, Pe and RTV.

	ITC					Pe					RTV				
***Coefficient***	*β*_*std*_	*SE*	*t-value*	*p*	*df*	*β*_*std*_	*SE*	*t-val*	*p*	*df*	*β*_*std*_	*SE*	*t-value*	*p*	*df*
**Intercept**	−0.01	0.08	−0.16	0.873	310.22	0.02	0.08	0.31	0.758	278.05	0.09	0.07	1.27	0.206	281.76
**PGS (Autism)**	0.00	0.05	0.08	0.933	364.90	0.07	0.05	1.26	0.208	292.82	0.03	0.05	0.52	0.607	304.27
**Age**	−0.00	0.06	−0.07	0.943	237.01	−0.03	0.06	−0.60	0.548	220.18	0.07	0.05	1.36	0.174	226.26
**Sex**	0.09	0.10	0.86	0.391	408.47	−0.04	0.11	−0.37	0.714	365.03	−0.24	0.10	−2.34	**0.020**	364.91
	R^2^ = 0.002; corrected R^2^ = 0.003					R^2^ = 0.005; corrected R^2^ = 0.008					R^2^ = 0.022; corrected R^2^ = 0.033				

Uncorrected p-values are given in the table, none of the p-values were lower than 0.05 after FDR correction. Only the fixed effect findings are given in the table. SE Standard Error; df degrees of freedom (Satterthwaite’s approximation).

*ITC* Inter-trial coherence, *Pe* Error positivity, *RTV* Reaction time variability.

**Table 5:** R^2^ for the models (${{\boldsymbol{R}}}_{{\boldsymbol{model}}}^{{\boldsymbol{2}}}$) in Tables 3 and 4, before and after reliability correction.

			Not reliability corrected			Reliability corrected		
PGS	Variable	*r* (test-retest)	${R}_{{model}}^{2}$	${R}_{{PGS}}^{2}$	β_std_ (95% CI)	${R}_{{model}}^{2}$	${R}_{{PGS}}^{2}$	β_std_ (95% CI)
**PGS ADHD**	**ITC**	0.777*	0.025*	0.024*	−0.156 (−0.259, −0.053)*	0.033*	0.031*	−0.177 (−0.294, −0.060)*
	**Pe**	0.711	0.001	0.000	0.017 (−0.090, 0.124)	0.001	0.000	0.020 (−0.107, 0.147)
	**RTV**	0.669	0.026	0.006	0.078 (−0.026, 0.182)	0.038	0.009	0.095 (−0.032, 0.223)
**PGS Autism**	**ITC**	0.777	0.002	0.000	0.004 (−0.099, 0.108)	0.003	0.000	0.005 (−0.112, 0.123)
	**Pe**	0.711	0.005	0.005	0.068 (−0.037, 0.173)	0.008	0.005	0.081 (−0.044, 0.205)
	**RTV**	0.669	0.022	0.001	0.027 (−0.076, 0.130)	0.033	0.001	0.033 (−0.093, 0.159)

Standardised beta coefficients for PGS of ADHD and PGS of autism (**β**_**std**_) and R^2^ estimated from the **β**_**std**_ of PGS alone ${{\boldsymbol{R}}}_{{\boldsymbol{PGS}}}^{{\boldsymbol{2}}}$ before and after reliability correction. The corrected value is the single corrected ${{\boldsymbol{\beta }}}_{{std}}/\sqrt{{r}_{{YY}}}$ where ${r}_{{YY}}$ is the test-retest correlation. ${{\boldsymbol{R}}}_{{\boldsymbol{PGS}}}^{{\boldsymbol{2}}}$ is calculated by squaring the **β**_**std**_ for PGS of ADHD and autism in Tables 3 and 4. * marks the statistically significant results for PGS.

*ITC* Inter-trial coherence, *Pe* Error positivity, *RTV* Reaction time variability.

Table 4 gives the associations between EEG measures (ITC and Pe amplitude) or RTV and PGS for autism. There was no statistically significant association between PGS for autism and ITC, Pe or RTV (Tables 4 and 5).

PGS for body mass index (BMI) and height were tested as negative controls for association with ITC, Pe and RTV. No significant associations were observed after correction for multiple comparisons (Supplementary Tables 4 and 5). The number of incongruent correct or incongruent incorrect trials was also tested for association with PGS of ADHD and autism. No statistically significant associations were found (Supplementary Tables 6 and 7).

## Discussion

In an investigation of the ability of PGS for ADHD and autism to predict cognitive control and reaction time measures previously showing genetic overlap with the conditions, we found that PGS for ADHD [41] predicts 2.5% of the total variance (corresponding to 3.3% of the reliable variance) in ITC of FMθ activity. This effect size is notable given that PGS typically explain 0.7% to 3.3% of variance in dimensional assessments of ADHD [66], suggesting that theta ITC may be a particularly sensitive indicator of genetic risk. Importantly, the genetic signal (2.4%) accounted for nearly all explained variance in the full model (2.5%) for theta ITC. In contrast, for RTV, sex explained the majority of model variance, with PGS contributing a smaller proportion. This finding aligns with previous research demonstrating shared genetic overlap between trial-to-trial phase variability in FMθ and ADHD in twin studies [15, 17]. The ADHD PGS score did not significantly predict other measures previously shown to have phenotypic and genetic overlap with ADHD, specifically RTV and the amplitude of the Pe, which indexes theta-related error processing. We also examined the predictive power of a PGS for autism on these measures, given the previously established genetic and phenotypic overlap between them and autism in twin studies. However, none of these measures showed significant prediction by the autism PGS.

The consistent links between ADHD and dysregulation in FMθ activity, together with genetic correlations from twin studies and the current PGS findings, provide robust evidence for associations between genetic variance, brain function and behaviour. The triangulation of evidence from cognitive experimental work [25, 29, 67, 68], multivariate twin analyses [15, 17], and now molecular genetic analysis provides robust support for the impact of this specific aspect of cognitive control in ADHD. An influential model of FMθ is that it communicates the need for behavioural control across different brain regions [20] facilitating information transfer via synchronised phase entrainment [4]. In this model, theta oscillations are proposed to signal the need for cognitive control from the anterior cingulate cortex to task-relevant neural networks, including sensory and motor regions [69, 70]. This signalling enables rapid and flexible neural responses to conflicting information or changing task demands [20, 71, 72]. Recent non-human primate research provides direct neural evidence for this mechanism, demonstrating that performance in cognitive control tasks varies systematically with the phase of frontal theta oscillations, with optimal performance occurring at specific phases [73]. In this context, the dysregulation of theta oscillations in ADHD may represent a mechanism for failure to implement and optimise task-relevant signalling across neural networks, ultimately leading to behavioural symptoms characteristic of the condition [4, 15, 17].

The PGS used in the present study was derived from DeMontis et al. (2019), the first report of genome-wide significant risk loci for ADHD [41]. In their original study, this PGS accounted for 5.5% of the variance in ADHD with a subsequent systematic review indicating the variance explained in dimensional assessments of ADHD is 0.7% to 3.3% [66]. Several of the genome-wide significant loci that informed the ADHD PGS have been implicated in neurodevelopment, regulation of synaptic homeostasis and functional cell to cell connections in the brain, including *FOXP2, SEMA6D* and *PCDH7* [74–76]. Another identified locus of the ADHD PGS contains DUSP6, a gene implicated in dopamine regulation [41]. This finding has clear relevance to ADHD as many effective ADHD medications target dopaminergic systems [77, 78] but it also may be relevant to FMθ activity, which has been shown to be diminished in a dopamine depletion rodent model [79].

To assess the measurement quality of the variables examined in our study, we calculated their test-retest reliability. This psychometric information allows us to correct for measurement error and more accurately estimate the true relationships between PGS and our phenotypic variables, potentially increasing our power to detect genetic associations. Moreover, test-retest reliability helps establish the upper limit of variance in the phenotype that could be explained by genetic factors as PGS cannot account for more variance than is reliably measured by the instrument. All cognitive control and reaction time measures derived from the arrow flanker task demonstrated moderate to excellent test-retest reliability. Among these, both theta ITC and the Pe exhibited excellent test-retest reliability: both 0.79 [62, 63]. These robust reliability estimates are consistent with a substantial body of previous research that has consistently shown good to excellent test-retest reliability for various EEG measures, including event-related potentials, spectral power, and connectivity metrics [80–83].

Several limitations of this study should be considered when interpreting our results. Our sample size may be insufficient to detect smaller genetic effects, and the varying reliability of our measures could impact our ability to detect genetic associations. These issues are illustrated by our findings regarding RTV and theta ITC. Despite previous twin analyses demonstrating a strong genetic overlap between RTV and ITC, we found a significant association between ADHD PGS and ITC, but not with RTV. This discrepancy could be due to our limited sample size lacking power to detect smaller effect sizes for RTV. Additionally, the lower test-retest reliability of RTV (0.61) compared to ITC (0.79) may have contributed to this result, as measurement error can attenuate genetic associations. Our sample was enriched for ADHD and autism trait variation, which may limit the generalisability of the findings, yet sensitivity analyses confirmed that the PGS–ITC association in this study remains robust in subsamples with population-representative ADHD prevalence and when examined in individuals without ADHD and those with ADHD separately. Furthermore, the current PGS captures only common genetic variants associated with ADHD, potentially missing other relevant genetic influences [84]. These factors collectively underscore the complexity of the relationships between genetic risk, brain function, and behaviour in ADHD. Our test-retest reliability estimates were based on a relatively small sample (n = 21), which results in wider confidence intervals and should be interpreted with caution, though the estimates for our key measures (ITC, Pe, RTV) were statistically significant and within ranges reported in previous EEG studies. An important limitation was that all participants were of white ethnic origin, which restricts the generalisability of our findings to other populations and underscores the necessity to include individuals from diverse ethnic backgrounds in research. Furthermore, ethnicity was recorded using broad categories at first contact, with ‘White’ not further subdivided, limiting assessment of within-group variation.

While the hypothesised link between irregular behavioural responses and inefficient signalling in frontal-midline theta remains compelling and is supported by extensive research [15, 17, 24–28], our results highlight the need for future studies with larger samples and more comprehensive genetic data. Such studies will be crucial to fully elucidate these intricate relationships and their role in ADHD aetiology, bridging the gap between behavioural genetics and molecular genetic approaches.

This is the first study to establish a relationship between the PGS for ADHD and FMθ ITC, providing a link between genetic risk factors for the condition and the irregular oscillatory dynamics that have been consistently linked with ADHD in cognitive and neurophysiological studies. The examination of precise neurophysiological measures such as FMθ ITC contributes to efforts to refine phenotype definition in genetic studies, potentially improving the robustness and replicability of findings in brain biomarker research.

This research advances our understanding of the genetic and neural underpinnings of ADHD, demonstrating the value of examining multiple levels of analysis in psychiatric research. The consistency of findings across methodological approaches—from twin studies to molecular genetics—provides a robust framework for understanding how impaired cognitive control, particularly as reflected in FMθ activity, contributes to the ADHD phenotype. Future research should focus on elucidating the precise mechanistic pathways through which genetic risk for ADHD shapes cognitive control processes at the neural level. By integrating genetic risk scores with reliable neural markers like theta ITC, this approach could advance the development of neurobiologically-informed diagnostic tools and enable stratification of patients based on their genetic and neural profiles, paving the way for more precise, personalised intervention strategies.

## Supplementary information

Supplemental Material

## Data Availability

The data that support the findings of this study are not publicly available due to ethical restrictions and participant consent limitations. Data from IDEAS may be made available upon reasonable request to the corresponding author, subject to appropriate ethical approval and data sharing agreements in accordance with institutional and regulatory requirements.
