# Supplementary material for: ADHD polygenic risk predicts neural signatures of cognitive control: Evidence from midfrontal theta dynamics
Source: Transl Psychiatry. 2026 Mar 31;16:174. doi: 10.1038/s41398-026-03938-2 (PMC13039785; doi:10.1038/s41398-026-03938-2)
Supplement: Supplementary file 1 — Supplemental Material [file 41398_2026_3938_MOESM1_ESM.docx]

**Supplemental Material**

**Title:** ADHD Polygenic Risk Predicts Neural Signatures of Cognitive Control: Evidence from Midfrontal Theta Dynamics

Running title: ADHD Polygenic Risk and Midfrontal Theta Dynamics

**Authors:**

**Ümit Aydin^1,2^, Ziye Wang^2,3^, Máté Gyurkovics**^4^**, Amy Tong**^5^**, Grace Cullen**^2,^**^6^, Sumayyah Ahmed**^2^**, Jason Palmer^7^,** Gráinne McLoughlin***^2^**

**Affiliations:**

^1^ School of Psychology & Clinical Language Sciences, University of Reading, Reading, United Kingdom

^2^ Social, Genetic & Developmental Psychiatry Centre, Institute of Psychiatry, Psychology & Neuroscience, King’s College London, London, United Kingdom

^3^ Department of Biological and Experimental Psychology, School of Biological and Behavioural Sciences, Queen Mary University of London, London, United Kingdom

^4^ School of Psychology, University of East Anglia, Norwich, United Kingdom

^5^ Department of Psychological Medicine, Institute of Psychiatry, Psychology & Neuroscience, King’s College London, London, United Kingdom

^6^ Faculty of Biology, Medicine and Health, University of Manchester, Manchester, United Kingdom

^7^ School of Mathematical and Data Sciences, West Virginia University, Morgantown, West Virginia, United States

* Corresponding author: grainne.mcloughlin@kcl.ac.uk

**Keywords:** EEG, Polygenic scores, ADHD, Autism, midfrontal theta, reaction time

**Participant recruitment strategy**

All participants were originally part of the Twins Early Development Study (TEDS; Haworth et al., 2013), a longitudinal cohort of twins born in England and Wales between 1994 and 1996. Participants were invited to take part in IDEAS through four recruitment routes designed to enrich for a wide range of ADHD and autism-related traits while retaining generalisability to the broader population. First, individuals were recruited from the *Social Relationships Study Phase 1*, which included twins identified in childhood as meeting diagnostic criteria for autism or displaying subclinical autistic traits, based on the Childhood Autism Spectrum Test (CAST) and follow-up Development and Wellbeing Assessment (DAWBA) interviews (Colvert et al., 2015). Control participants in this group were matched on demographics but had low CAST scores in childhood. Second, participants were drawn from the *Neurophysiological Study of Activity and Attention in Twins (NEAAT)*, a TEDS subsample enriched for ADHD traits based on longitudinal symptom trajectories from childhood to adolescence (Tye et al., 2012). Third, participants were recruited from *Social Relationships Study Phase 3*, which included new participants not part of the original SR study but who had either a formal autism diagnosis or high current and past autistic traits. Finally, additional participants were sampled from the broader *TEDS main cohort*, including those with stably high or low ADHD traits across development, as well as controls with low CAST scores and adequate estimated IQ. Medical exclusions followed TEDS protocol and included severe ASD, global developmental delay, cerebral palsy, and other significant neurodevelopmental conditions (Haworth et al., 2013). Detailed recruitment figures and selection criteria for each route are reported in Capp et al. (2022).

**EEG processing and analysis**

Following average referencing, Adaptive Mixture ICA (AMICA) was used to calculate ICA components (Palmer et al., 2011) using the nsgportal plug-in on the high-performance computing resources available on Neuroscience Gateway (NSG, nsgportal.org) (Martínez-Cancino et al., 2021). The ICLabel algorithm was used for automatic detection and removal of ICs representing ocular artefacts (3). Continuous data was divided into epochs. Incongruent-incorrect trials were response-locked to incorrect responses with epochs -900 to 600 ms based on the time of the button press. Congruent-correct and incongruent-correct trials were stimulus-locked to correctly answered stimuli with epochs from -500 ms to 1000 ms relative to the onset of the target stimulus. All trials were baseline corrected using baseline -900 ms to -600 ms for response-locked and -400 ms to -100 ms for stimulus-locked trials.

**Genotyping and polygenic score calculation**

Genotyping for the Twins Early Development Study (TEDS) participants was performed in two phases, using two platforms: the Affymetrix GeneChip 6.0 array and the Illumina HumanOmniExpressExome-8v1.2 array. Following standard quality control procedures, SNPs with low call rates (< 98%), deviations from Hardy-Weinberg equilibrium (p < 10^-5^), or minor allele frequency (MAF) < 0.5% were excluded. Genotypes were phased using EAGLE v2 and imputed into the Haplotype Reference Consortium (release 1.1) via the Sanger Imputation Service. After imputation, SNPs with an INFO score < 0.75 were removed, yielding approximately 7.36 million high-quality markers. To ease high computational demands of the software that generates polygenic scores, SNPs with INFO < 1 were further excluded, leaving 515,000 SNPs for analysis.

Polygenic scores were generated using LDpred v1.0.6, assuming a prior on the fraction of causal markers of 1 (i.e., an infinitesimal model). Scores were calculated as the sum of the imputed risk allele dosages weighted by the SNP effect sizes from the discovery genome-wide association study (GWAS). Polygenic scores were residualised for the first ten principal components of ancestry, genotyping chip, and batch, and then standardised (mean = 0, SD = 1) prior to analysis. Detailed information on genotyping, imputation, and polygenic score construction in TEDS is available in Selzam et al. (2019).

**Supplementary Table 1:** Reliability estimates and standard errors of EEG and behavioural measures calculated from the arrow flanker task

| Variable | ICC (95% CI) | Pearson’s *r* (95% CI) | ICC SE | Pearson’s *r SE* |
| --- | --- | --- | --- | --- |
| ITC | .785 (.540, .907) | .777 (.520, .905) | .09 | .10 |
| Pe amplitude | .791 (.486, .915) | .711 (.403, .874) | .11 | .12 |
| RTV | .605 (.227, .821) | .669 (.334, .854) | .15 | .13 |
| ERN amplitude | .552 (-.015, .812) | .426 (-.007, .724) | .21 | .19 |
| N2 Incongruent amplitude | .648 (.124, .858) | .472 (.050, .751) | .19 | .18 |
| N2 Congruent amplitude | .599 (.007, .838) | .433 (.001, .728) | .21 | .19 |
| RTM | .859 (.522, .950) | .891 (.745, .955) | .11 | .05 |
| Pe latency | .505 (-.113, .791) | .374 (-.069, .694) | .23 | .20 |
| ERN latency | .813 (.550, .924) | .692 (.371, .865) | .10 | .13 |
| N2 Incongruent latency | .678 (.235, .867) | .572 (.187, .805) | .16 | .16 |
| N2 Congruent latency | .447 (-.305, .771) | .417 (-.018, .719) | .27 | .19 |

ERN: Error-related negativity, N2 Incongruent: N2 of incongruent trials, N2 Congruent.: N2 of Congruent trials, RTM: Mean reaction time, Pe: Error positivity


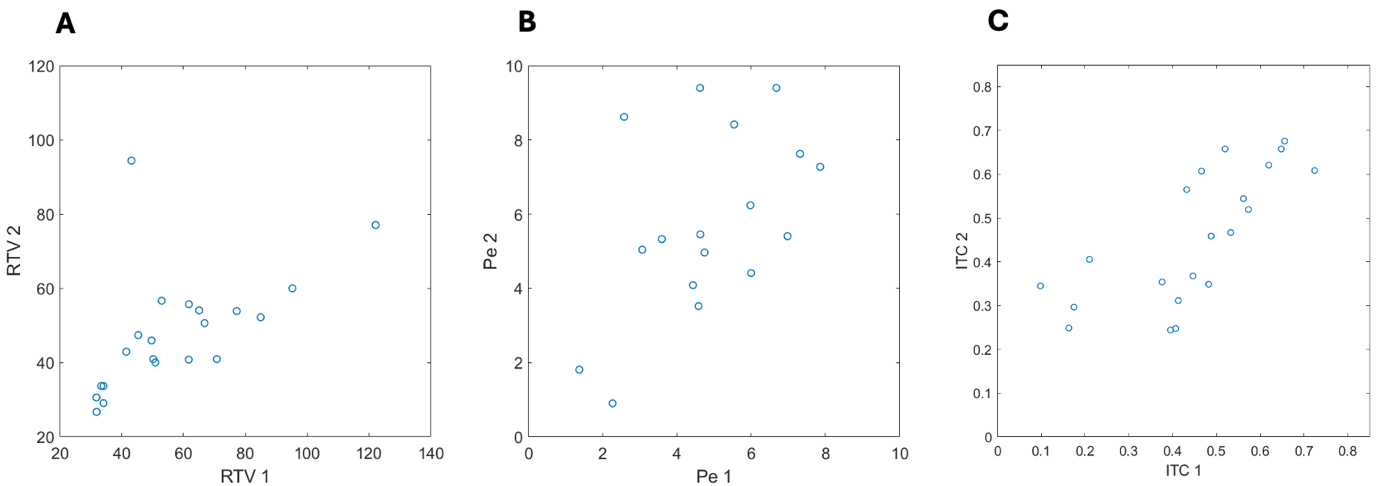


**Supplementary Figure 1: Scatter plots of test-retest data for ITC (A), Pe (B) and RTV (C).**

**Supplementary Table 2:** Associations between the PGS of ADHD and childhood Conners’ Parent Rating Scale (CPRS), total adulthood ADHD symptoms in DIVA and adulthood ADHD (DIVA diagnosis), and uncorrected p-values that were lower than 0.05 are indicated in BOLD and the ones lower than 0.05 after FDR correction are indicated with *. Only the variables of interest, PGS, were corrected for multiple comparisons for the three models. For the adulthood ADHD generalized linear mixed model, as implemented in glmer, was used with a logistic link function. Only the fixed effect findings are given in the table. SE: Standard Error; df: degrees of freedom (Satterthwaite’s approximation).

|  | **Childhood CPRS** | | | | | **ADHD Total Symptoms** | | | | | | **ADHD (DIVA diagnosis)** | | | | | |  |
| --- | --- | --- | --- | --- | --- | --- | --- | --- | --- | --- | --- | --- | --- | --- | --- | --- | --- | --- |
| ***Coefficient*** | *β_std_* | *SE* | *t-value* | *p* | *df* | | *β_std_* | *SE* | *t-value* | *p* | *df* | | *Odds ratios* | *SE* | *z-value* | *p* | *Df* | |
| **Intercept** | -0.30 | 0.07 | -4.10 | **<.001** | 299.74 | | -0.15 | 0.07 | -2.11 | **0.036** | 313.77 | | 0.10 | 0.03 | -6.55 | **<.001** | Inf | |
| **PGS (ADHD)** | 0.19 | 0.05 | 3.95 | **<.001*** | 379.01 | | 0.07 | 0.05 | 1.35 | 0.177 | 362.32 | | 1.23 | 0.20 | 1.28 | 0.201 | Inf | |
| **Age** | -0.10 | 0.06 | -1.80 | .073 | 215.95 | | -0.06 | 0.05 | -1.10 | 0.273 | 240.06 | | 0.74 | 0.12 | -1.77 | 0.077 | Inf | |
| **Sex** | 0.50 | 0.09 | 5.38 | **<.001** | 411.90 | | 0.24 | 0.09 | 2.53 | **0.012** | 428.85 | | 1.99 | 0.64 | 2.16 | **0.031** | Inf | |
| R^2^= .106 | | | | | | R^2^= .025 | | | | | | R^2^= .051 | | | | | |  |

**Supplementary Table 3:** Associations between the PGS of Autism and childhood Asperger Syndrome Test (CAST; measured at age 11.09 ± 0.74 years), adult ADOS total score. The analysis for adult Autism (ADOS diagnosis) are not presented because the model failed to converge. Only the fixed effect findings are given in the table. SE: Standard Error; df: degrees of freedom (Satterthwaite’s approximation)

|  | **Childhood CAST** | | | | | **ADOS total score** | | | | | | |  |  |
| --- | --- | --- | --- | --- | --- | --- | --- | --- | --- | --- | --- | --- | --- | --- |
| ***Coefficient*** | *β_std_* | *SE* | *t-value* | *p* | *df* | | *β_std_* | *SE* | *t-value* | *p* | *df* | | |  |
| **Intercept** | -0.27 | 0.07 | -3.83 | **<.001** | 284.64 | | -0.23 | 0.07 | -3.48 | .001 | | 311.99 | | |
| **PGS (Autism)** | -0.02 | 0.05 | -0.33 | .742 | 324.15 | | -0.05 | 0.05 | -1.06 | .292 | | 355.65 | | |
| **Age** | -0.02 | 0.05 | -0.38 | 0.702 | 215.60 | | 0.00 | 0.05 | 0.02 | .984 | | 241.51 | | |
| **Sex** | 0.41 | 0.10 | 4.24 | **<0.001** | 390.90 | | 0.32 | 0.09 | 3.58 | **<0.001** | | 419.60 | | |
| R^2^= .045 | | | | | | R^2^= .033 | | | | | | |  |  |

**Supplementary Table 4:** Associations between the PGS of body mass index (BMI) and ITC, Pe and RTV. Age and Sex were added as covariates. Uncorrected p-values are given in the table, none of the p-values were lower than 0.05 after FDR correction. FDR correction was considering the six models in Suppl. Table 4 and Suppl. Table 5. Only the fixed effect findings are given in the table. SE: Standard Error; df: degrees of freedom (Satterthwaite’s approximation)

|  | **ITC** | | | | | **Pe** | | | | | **RTV** | | | | |  |
| --- | --- | --- | --- | --- | --- | --- | --- | --- | --- | --- | --- | --- | --- | --- | --- | --- |
| ***Coefficient*** | *β_std_* | *SE* | *t-value* | *p* | *df* | *β_std_* | *SE* | *t-value* | *p* | *df* | *β_std_* | *SE* | *t-value* | *p* | *df* | |
| **Intercept** | -0.01 | 0.08 | -0.08 | .934 | 311.30 | 0.03 | 0.08 | 0.34 | .731 | 278.21 | 0.09 | 0.07 | 1.19 | .235 | 281.83 | |
| **PGS (BMI)** | -0.12 | 0.05 | -2.24 | **.026** | 370.00 | -0.11 | 0.05 | -1.94 | .053 | 305.19 | 0.11 | 0.05 | 2.07 | **.039** | 303.65 | |
| **Age** | -0.00 | 0.06 | -0.00 | .997 | 237.50 | -0.03 | 0.06 | -0.49 | .628 | 220.50 | 0.07 | 0.05 | 1.33 | .183 | 227.14 | |
| **Sex** | 0.07 | 0.10 | 0.70 | .481 | 408.20 | -0.05 | 0.11 | -0.43 | .666 | 363.82 | -0.23 | 0.10 | -2.19 | **.029** | 360.89 | |
|  | R^2^= .016 | | | | R^2^= .011 | | | | |  | R^2^= .032 | | | | | |

ITC: Inter-trial coherence, Pe: Error positivity, RTV: Reaction time variability

**Supplementary Table** 5**:** Associations between the PGS of height and ITC, Pe and RTV. Age and Sex were added as covariates. Uncorrected p-values are given in the table, none of the p-values were lower than 0.05 after FDR correction. FDR correction was considering the six models in Suppl. Table 4 and Suppl. Table 5. Only the fixed effect findings are given in the table. SE: Standard Error; df: degrees of freedom (Satterthwaite’s approximation)

|  | **ITC** | | | | | **Pe** | | | | | **RTV** | | | | |  |
| --- | --- | --- | --- | --- | --- | --- | --- | --- | --- | --- | --- | --- | --- | --- | --- | --- |
| ***Coefficient*** | *β_std_* | *SE* | *t-value* | *p* | *df* | *β_std_* | *SE* | *t-value* | *p* | *df* | *β_std_* | *SE* | *t-value* | *p* | *df* | |
| **Intercept** | -0.01 | 0.08 | -0.16 | .872 | 310.43 | 0.02 | 0.08 | 0.30 | .767 | 279.21 | 0.09 | 0.07 | 1.28 | .201 | 278.90 | |
| **PGS (height)** | 0.02 | 0.05 | 0.43 | .666 | 337.83 | -0.03 | 0.05 | -0.60 | .552 | 282.35 | -0.11 | 0.05 | -2.15 | **.033** | 276.69 | |
| **Age** | -0.00 | 0.06 | -0.04 | .964 | 236.39 | -0.03 | 0.06 | -0.57 | .568 | 221.00 | 0.07 | 0.05 | 1.28 | .202 | 223.96 | |
| **Sex** | 0.09 | 0.10 | 0.86 | .391 | 408.47 | -0.03 | 0.11 | -0.32 | .747 | 364.85 | -0.24 | 0.10 | -2.32 | **.021** | 359.26 | |
|  | R^2^= .002 | | | | R^2^= .002 | | | | |  | R^2^= .033 | | | | | |

ITC: Inter-trial coherence, Pe: Error positivity, RTV: Reaction time variability

**Supplementary Table 6:** Associations between the PGS of ADHD and the number of valid incongruent correct and incongruent incorrect trials. Age and Sex were added as covariates. Uncorrected p-values are given in the table and none of the p-values for PGS were lower than 0.05 after FDR correction. Only the fixed effect findings are given in the table. SE: Standard Error; df: degrees of freedom (Satterthwaite’s approximation)

|  | **Number of incongruent correct trials** | | | | | **Number of incongruent incorrect trials** | | | | |
| --- | --- | --- | --- | --- | --- | --- | --- | --- | --- | --- |
| ***Coefficient*** | *β_std_* | *SE* | *t-value* | *p* | *df* | *β_std_* | *SE* | *t-value* | *p* | *df* |
| **Intercept** | 102.04 | 27.24 | 3.75 | **<0.001** | 224.60 | 85.94 | 21.95 | 3.91 | **<0.001** | 217.67 |
| **PGS (ADHD)** | -0.11 | 1.17 | -0.09 | 0.925 | 288.30 | -0.93 | 0.93 | -0.99 | 0.323 | 294.26 |
| **Age** | 2.12 | 1.21 | 1.75 | 0.081 | 223.59 | -1.90 | 0.97 | -1.95 | 0.052 | 216.61 |
| **Sex** | 0.81 | 2.34 | 0.35 | 0.728 | 340.00 | 1.60 | 1.84 | 0.87 | 0.386 | 353.69 |
|  | R^2^= .008 | | | | R^2^=.016 | | | | |  |

**Supplementary Table 7:** Associations between the PGS of Autism and the number of valid incongruent correct and incongruent incorrect trials. Age and Sex were added as covariates. Uncorrected p-values are given in the table and none of the p-values for PGS were lower than 0.05 after FDR correction. Only the fixed effect findings are given in the table. SE: Standard Error; df: degrees of freedom (Satterthwaite’s approximation)

|  | **Number of incongruent correct trials** | | | | | **Number of incongruent incorrect trials** | | | | |
| --- | --- | --- | --- | --- | --- | --- | --- | --- | --- | --- |
| ***Coefficient*** | *β_std_* | *SE* | *t-value* | *p* | *df* | *β_std_* | *SE* | *t-value* | *p* | *df* |
| **Intercept** | -0.00 | 0.06 | -0.04 | **.972** | 274.98 | -0.01 | 0.07 | -0.16 | **.872** | 275.52 |
| **PGS (Autism)** | 0.00 | 0.05 | 0.06 | .952 | 281.37 | -0.01 | 0.05 | -0.28 | .777 | 288.36 |
| **Age** | 0.08 | 0.05 | 1.74 | .082 | 223.24 | -0.10 | 0.05 | -1.97 | **.05** | 216.97 |
| **Sex** | 0.03 | 0.09 | 0.34 | .733 | 341.57 | 0.08 | 0.10 | 0.85 | .393 | 355.88 |
|  | R^2^= .008 | | | | R^2^=.014 | | | | |  |

**Supplementary Table 8:** Task performance statistics for the arrow flanker task. Mean ± std are given, and the range is given in parentheses below.

|  | Correct | Incorrect | Omitted |
| --- | --- | --- | --- |
| Congruent | 190.37 ± 21.27  (32 - 272) | 4.45 ± 10.79  (0 – 161) | 1.50 ± 4.82  (0 – 58) |
| Incongruent | 150.57 ± 23.38  (41 – 228) | 43.95 ± 18.56  (4 – 125) | 1.83 ± 4.93  (0 – 59) |

**Supplementary Table 9:** Sensitivity analyses testing the association between ADHD polygenic score (PGS) and intertrial coherence (ITC). We conducted three sensitivity analyses: (1) random subsampling (1,000 iterations) approximating population ADHD prevalence (3% ADHD, 97% controls; n = 347 per iteration) through subsampling ADHD individuals, (2) analysis restricted to control participants only (n = 337), and (3) analysis restricted to participants with ADHD only (n = 77). All analyses used the same mixed-effects model structure as the main analysis, controlling for age, sex, and family clustering.

|  | n | Βstd | t-value | p-value | R^2^ |
| --- | --- | --- | --- | --- | --- |
| Population-representative (3% ADHD)ᵃ | 347 | -0.125 [-0.143, -0.112] | -2.22^b^ | .029^c^ | .020 |
| ****Controls only**** | 337 | -0.119 | -2.09 | .038 | .020 |
| ****ADHD only**** | 77 | -0.291 | -2.29 | .025 | .068 |

a Ayano et al., 2024; Song et al. 2021; NICE, 2025

b Mean t-value across 1,000 iterations. 95% CI: [-2.56, -1.97].

b Mean p-value across 1,000 iterations. 95% CI: [0.011, 0.050]. 97.5% of iterations showed p < .05.

**References**

Ayano, G., Tsegay, L., Gizachew, Y., Necho, M., Yohannes, K., Demelash, S., Anbesaw, T., & Alati, R. (2024). Prevalence of ADHD in Adults: An Umbrella Review of International Studies. European Psychiatry, 67(S1), S343.

Capp, S. J., Agnew-Blais, J., Lau-Zhu, A., Colvert, E., Tye, C., Aydin, Ü., ... & McLoughlin, G. (2023). Is quality of life related to high autistic traits, high ADHD traits and their interaction? Evidence from a young-adult community-based twin sample. *Journal of Autism and Developmental Disorders*, *53*(9), 3493-3508.

Colvert, E., Tick, B., McEwen, F., Stewart, C., Curran, S. R., Woodhouse, E., Gillan, N., Hallett, V., Lietz, S., Garnett, T., Ronald, A., Plomin, R., Rijsdijk, F., Happé, F., & Bolton, P. (2015). Heritability of autism spectrum disorder in a UK population-based twin sample. *JAMA Psychiatry*, 72(5), 415–423

Haworth, C. M. A., Davis, O. S. P., & Plomin, R. (2013). Twins Early Development Study (TEDS): A genetically sensitive investigation of cognitive and behavioural development from childhood to young adulthood. *Twin Research and Human Genetics*, 16(1), 117–125.

Martínez-Cancino R, Delorme A, Truong D, Artoni F, Kreutz-Delgado K, Sivagnanam S, et al. (2021): The open EEGLAB portal Interface: High-Performance computing with EEGLAB. NeuroImage 224: 116778.

NICE Clinical Knowledge Summaries (2025). Attention deficit hyperactivity disorder. National Institute for Health and Care Excellence.

Palmer JA, Kreutz-Delgado K, Makeig S (2011): AMICA: An Adaptive Mixture of Independent Component Analyzers with Shared Components. 15.

Selzam, S., Ritchie, S. J., Pingault, J.-B., Reynolds, C. A., O'Reilly, P. F., & Plomin, R. (2019). Comparing Within- and Between-Family Polygenic Score Prediction. *American Journal of Human Genetics*, 105(2), 351–363.

Song, P., Zha, M., Yang, Q., Zhang, Y., Li, X., Rudan, I., & the Global Health Epidemiology Reference Group (GHERG) (2021). The prevalence of adult attention-deficit hyperactivity disorder: A global systematic review and meta-analysis. Journal of Global Health, 11, 04009.

Tye, C., Rijsdijk, F., Greven, C. U., Kuntsi, J., Asherson, P., & McLoughlin, G. (2012). Shared genetic influences on ADHD symptoms and very low-frequency EEG activity: A twin study. *Journal of Child Psychology and Psychiatry*, 53(6), 706–715.
